# A VEGF-A/SOX2/SRSF2 network controls *VEGFR1* pre-mRNA alternative splicing in lung carcinoma cells

**DOI:** 10.1038/s41598-018-36728-y

**Published:** 2019-01-23

**Authors:** Cherine Abou Faycal, Sylvie Gazzeri, Beatrice Eymin

**Affiliations:** 1 INSERM U1209, CNRS UMR5309, Institute For Advanced Biosciences, Grenoble, 38042 France; 2 Université Grenoble Alpes, Institut Albert Bonniot, Grenoble, 38041 France

## Abstract

The splice variant *sVEGFR1-i13* is a truncated version of the cell membrane-spanning VEGFR1 receptor that is devoid of its transmembrane and tyrosine kinase domains. We recently showed the contribution of sVEGFR1-i13 to the progression and the response of squamous lung carcinoma to anti-angiogenic therapies. In this study, we identify VEGF165, a splice variant of VEGF-A, as a regulator of sVEGFR1-i13 expression in these tumors, and further show that VEGF_165_ cooperates with the transcription factor SOX2 and the splicing factor SRSF2 to control sVEGFR1-i13 expression. We also demonstrate that anti-angiogenic therapies up-regulate sVEGFR1-i13 protein level in squamous lung carcinoma cells by a mechanism involving the VEGF_165_/SOX2/SRSF2 network. Collectively, our results identify for the first time a signaling network that controls *VEGFR1* pre-mRNA alternative splicing in cancer cells.

## Introduction

Neo-angiogenesis is the formation of new blood vessels from pre-existing ones that contribute to tumor oxygenation and nutrients supply during carcinogenesis. At the molecular level, this process requires the binding of VEGF-A to vascular endothelial growth factor receptors 1 (VEGFR1) and 2 (VEGFR2) and downstream activation of various signaling pathways including PI3K/AKT or MAPK. This leads to endothelial cells proliferation, survival, adhesion and/or migration and the formation of new vessels from pre-existing ones^1,2^. The VEGF-A/VEGFR network is subjected to various regulations including transcriptional and post-transcriptional mechanisms. Hence, in addition to the transmembrane VEGFR1, soluble isoforms of the receptor (sVEGFR1s) which arise from cleavage of full-lenght VEGFR1 or from alternative splicing of *VEGFR1* pre-mRNA are produced by endothelial and also tumor cells. sVEGFR1s have been implicated in many pathological functions such as tumor progression^3,4^. In addition, several clinical trials have shown that anti-angiogenic therapies up-regulate circulating levels of sVEGFR1s^5–7^. However, the molecular determinants that control the expression of sVEGFR1s in cancer remain largely unknown.

**Figure 1:**
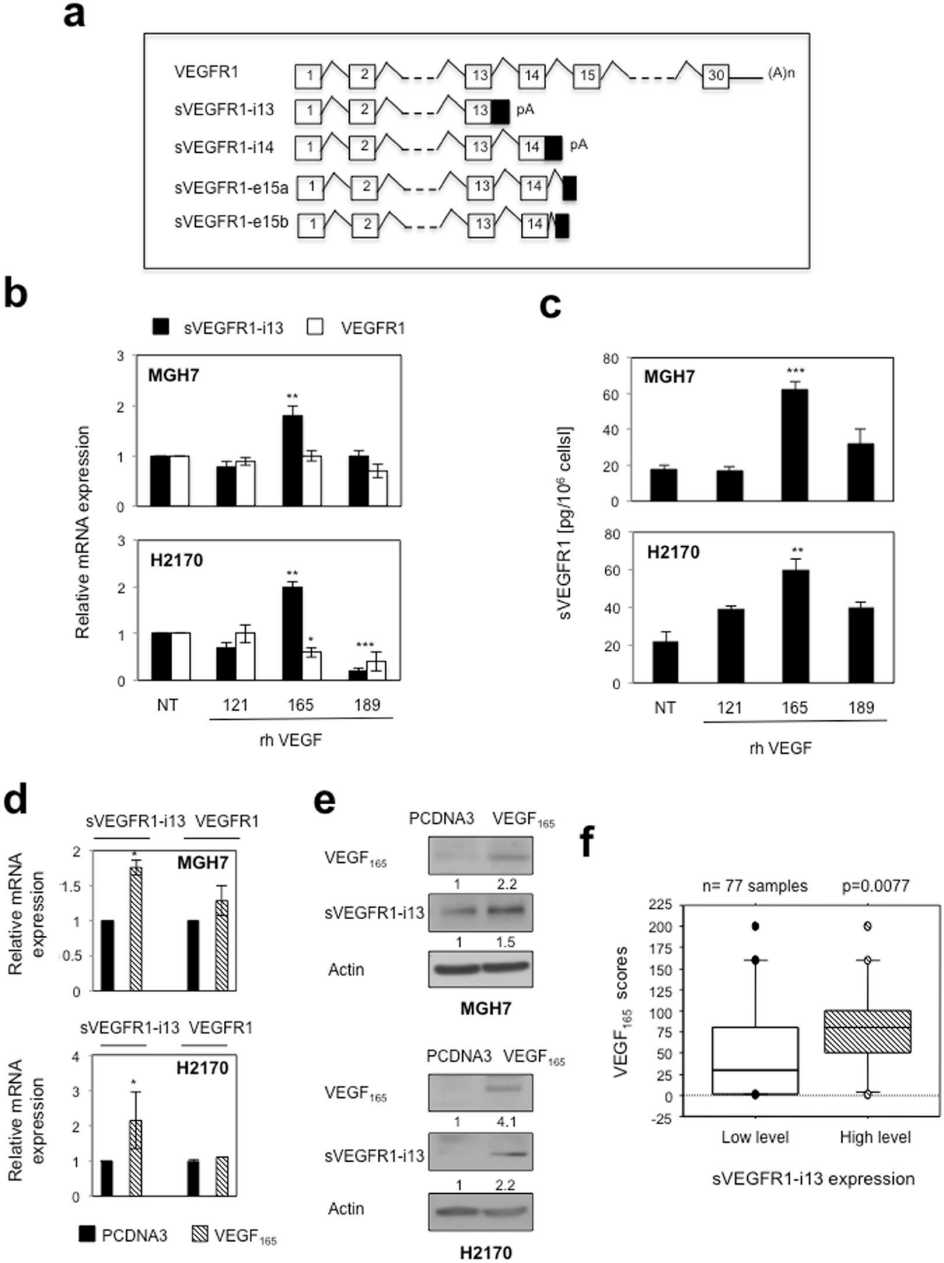
VEGF_165_ regulates sVEGFR1-i13 expression in SQLC cell lines. (**a**) Schematic representation of the full-length *VEGFR1* transcript and the different *sVEGFR1* splice variants. (**b**,**c**) MGH7 (upper histogram) and H2170 (lower histogram) cells treated or not (NT) with 1 ng/ml rhVEGF_121_, rhVEGF_165_ or rhVEGF_189_ during 24 hours. (**b**) RT-qPCR analyses of *sVEGFR1-i13* or *VEGFR1*. *GAPDH* was used as an internal control. The value 1 was arbitrarily assigned to the untreated condition signal. (**c**) ELISA assays for quantification of sVEGFR1-i13 in the cell pellets. (**d**,**e**) MGH7 and H2170 cells were transfected with pcDNA3 or pcDNA3-VEGF_165_ plasmid for 48 hours. (**d**) RT-qPCR analyses of *sVEGFR1-i13* and *VEGFR1*. *GAPDH* was used as an internal control. (**e**) Western-blot analyses of VEGF_165_ and sVEGFR1-i13 in MGH7 or H2170 cells as indicated. Actin was used as a loading control. Numbers represent the quantification of VEGF_165_ or sVEGFR1-i13 signal intensities relative to actin signal using Image J software. The value 1 was arbitrarily assigned to the pcDNA3 condition signal. All western blot experiments were performed at least three times. Illustrations of a representative result are presented for each condition. (**f**) Mean levels ± SD of VEGF_165_ immunohistochemical scores according to sVEGFR1-i13 status in squamous cell lung carcinoma, where SQLC are sub-divided in two classes representing tumors with high or low levels of sVEGFR1-i13 compared to normal lung tissues^4^. Statistical analyses were performed using a non parametric Mann-Whitney test (*p < 0.05; **p < 0.01; ***p < 0.001).

Four *VEGFR1* splice variants have been described to date, namely *sVEGFR1-i13*, *sVEGFR1-i14*, *sVEGFR1-e15a* and *sVEGFR1-e15b*. They are all common through to exon 13 but differ in their unique C-terminus (Fig. 1a)^8^. Among these splice variants, *sVEGFR1-i13* derives from intron 13 retention followed by premature polyadenylation^9^. sVEGFR1-i13 comprises the first six Ig-like domains of the extra-cellular region of the receptor, a specific 31 amino acids C-terminal tail and is devoid of the transmembrane and tyrosine kinase domains of full lenght VEGFR1. At the functional level, sVEGFR1-i13 is mainly viewed as a natural VEGF-A antagonist which inhibits the mitogenic effects of this growth factor by functioning as a dominant-negative trapping protein^10^ or by forming non-signaling complexes with VEGFR2^11^. sVEGFR1-i13 is therefore considered as an inhibitor of neo-angiogenesis which prevents tumor growth and metastasis in mouse models^12^. Conversely, it has been shown that sVEGFR1-i13 is part of the extracellular matrix and mediates the adhesion and migration of endothelial cells through direct binding to α5β1 integrin^13,14^. Together, these data support the notion that sVEGFR1-i13 exerts both pro- and anti-angiogenic functions on endothelial cells. Interestingly, we recently demonstated that sVEGFR1-i13 contributes to the progression and the response of Squamous Lung Carcinoma (SQLC) cells to anti-angiogenic therapies through the regulation of a β1 integrin/VEGFR autocrine loop^4^. Therefore, these data indicated that sVEGFR1-i13 also targets the tumor cells themselves.

In endothelial cells, several signals controlling sVEGFR1-i13 expression have been identified. It has been shown that VEGF-A upregulates sVEGFR1-i13 level by a mechanism depending on VEGFR2^15,16^. A cooperative role between the arginine demethylase and lysine hydroxylase JMJD6 (JuMonJi Domain containing-protein 6) and the splicing factor U2AF65 was also reported to control sVEGFR1-i13 expression^17^. Moreover, a NOTCH1 decoy variant which reduces NOTCH1 signaling was shown to increase sVEGFR1-i13 levels and to inhibit angiogenesis in retinas and tumors^18^. Up to now, the molecular mechanisms that regulate sVEGFR1-i13 expression in cancer cells have not been described. In this study, we identify a VEGF_165_/SOX2/SRSF2 network that controls sVEGFR1-i13 expression in squamous lung carcinoma cells. Importantly, this network also contributes to sVEGFR1-i13 accumulation in response to anti-angiogenic therapies.

## Results

### VEGF_165_ controls sVEGFR1-i13 expression in lung cancer cells

It was previously shown that VEGF-A up-regulates sVEGFR1-i13 but not full-lenght VEGFR1 expression in human vascular endothelial cells^16^. To test whether VEGF-A controls sVEGFR1-i13 in tumor cell lines, we treated MGH7 and H2170 squamous lung carcinoma cells for 24 hours with various recombinant splice variants of VEGF-A, namely rhVEGF_121_, rhVEGF_165_ and rhVEGF_189_. In both cell lines, an increase of *sVEGFR1-i13* but not *VEGFR1* mRNA level was observed upon treatment with rhVEGF_165_ only (Fig. 1b). Similar results were obtained when sVEGFR1 levels were quantified by ELISA assay in cellular extracts (Fig. 1c). To confirm these data, we transiently transfected MGH7 and H2170 cells with a plasmid encoding VEGF_165_. We showed that *sVEGFR1-i13* mRNA (Fig. 1d) and protein (Fig. 1e) levels are upregulated in cells overexpressing VEGF_165_ as compared to control cells. In contrast, full-lenght *VEGFR1* mRNA was not affected by VEGF_165_ (Fig. 1d). Taken together, these data demonstrated that VEGF_165_ regulates *VEGFR1* pre-mRNA splicing in favor of its truncated splice variant *sVEGFR1-i13* in lung tumor cells. To confirm the link between VEGF_165_ and sVEGFR1-i13, we took advantage of a retrospective Non Small Cell Lung Carcinoma (NSCLC) cohort in which we previously performed VEGF_165_, VEGFR1 and sVEGFR1-i13 immunohistochemical stainings^4,19^. In agreement with our results in cell lines, NSCLC patients with high VEGF_165_ scores were those with high sVEGFR1-i13 level (Fig. 1f, p = 0.007). Of note, no relationship between VEGF_165_ scores and VEGFR1 immunostainings was observed in these patients (data not shown).

### VEGF_165_ cooperates with SOX2 to regulate sVEGFR1-i13 expression in lung cancer cells

**Figure 2:**
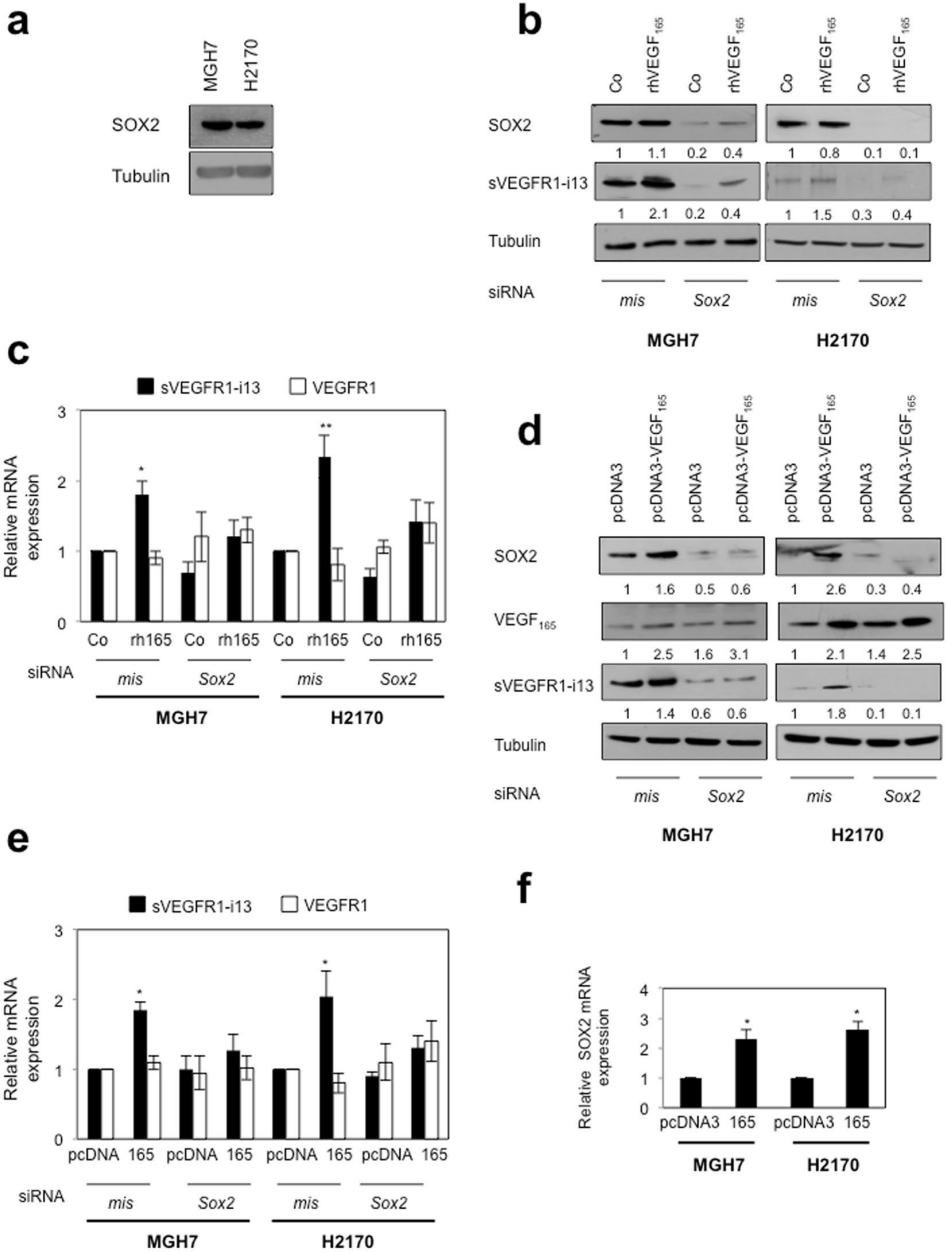
VEGF_165_ and SOX2 control sVEGFR1-i13 expression in SQLC cell lines. (**a**,**b**,**d**) Western blot analyses of SOX2, VEGF_165_ and/or sVEGFR1-i13 proteins were performed in MGH7 or H2170 cells either untransfected (**a**), or transfected with *mismatch* (mis) or *Sox2* (Sox2) siRNA during 48 hours and treated or not (Co) for 24 additional hours with 1 ng/ml rhVEGF_165_ (**b**), or co-transfected during 72 hours with *mismatch* (mis) or *Sox2* (Sox2) siRNA in the presence of a pcDNA3 or pcDNA3-VEGF_165_ plasmid for 48 hours (**d**). Tubulin was used as a loading control. Numbers represent the quantification of SOX2, sVEGFR1-i13 or VEGF_165_ signal intensities relative to tubulin signal using Image J software. The value 1 was arbitrarily assigned to the untreated condition signal. All western blot experiments were performed at least three times. Illustrations of a representative result are presented for each condition. (**c**,**e**) RT-qPCR analyses of *sVEGFR1-i13* or *VEGFR1* mRNA level were performed in cells treated in the same conditions as in b (**c**) or d (**e**). *GAPDH* was used as an internal control. The value 1 was arbitrarily assigned to the untreated condition signal. (**f**) RT-qPCR analyses of *SOX2* mRNA level in MGH7 or H2170 cells transfected with pcDNA3 or pcDNA3-VEGF_165_ (165) plasmid for 48 hours. *GAPDH* was used as an internal control. The value 1 was arbitrarily assigned to the control condition signal.

It was recently reported that VEGF-A controls the expression of the transcription factor SOX2 in breast and lung cancer cells^20^. As SOX2 is amplified in about 30% of squamous lung carcinoma patients^21^, we asked whether it plays a role in the regulation of sVEGFR1-i13 by VEGF_165_. We first showed that MGH7 and H2170 cells express detectable levels of SOX2 protein (Fig. 2a). When SOX2 was neutralized by siRNA, the increase of sVEGFR1-i13 mRNA and protein levels following treatment with rhVEGF_165_ (Fig. 2b,c) or after transfection with a plasmid encoding VEGF_165_ (Fig. 2d,e) was prevented. *VEGFR1* mRNA levels were never affected by the knock-down of SOX2, whatever the conditions. Interestingly, a decrease of sVEGFR1-i13 protein level was observed when SOX2 was neutralized (Fig. 2b,d), thereby indicating that SOX2 might also control sVEGFR1-i13 expression in the absence of VEGF_165_ stimulation. We noticed an increase of SOX2 protein (Fig. 2d) and mRNA (Fig. 2f) levels in cells transfected with the plasmid encoding VEGF_165_, consistent with previous data demonstrating that VEGF-A controls SOX2 expression in lung cancer cells^20^. As a whole, our data provided the first evidence that a VEGF_165_/SOX2 signaling network regulates sVEGFR1-i13 expression in squamous lung carcinoma cells.

In SQLC patients, inhibitory alterations of NOTCH signaling are frequent. In addition, in transgenic lung tumor mouse models, SOX2 binds to *Notch1* and *Notch2* regulatory regions leading to a significant reduction of *Notch1* and *Notch2* transcripts^22^. As it was recently shown that inhibition of NOTCH signaling by a NOTCH1 decoy variant increases sVEGFR1-i13 level in endothelial cells^18^, we thus tested whether NOTCH signaling regulates sVEGFR1-i13 expression in SQLC cells. To do so, MGH7 or H2170 cells were treated with FLI-06, a gamma-secretase inhibitor which blocks NOTCH processing and trafficking. As compared to control untreated cells, we did not observe any significant variation in sVEGFR1-i13 protein (Supplementary Fig. 1a), or mRNA (Supplementary Fig. 1b), o) levels. Rather a global increase in both *VEGFR1* and *sVEGFR1-i13* mRNA levels was observed following FLI-06 treatment (Supplementary Fig. 1b).

### A VEGF_165_/SOX2/SRSF2 signaling network controls sVEGFR1-i13 expression in lung tumor cells

**Figure 3:**
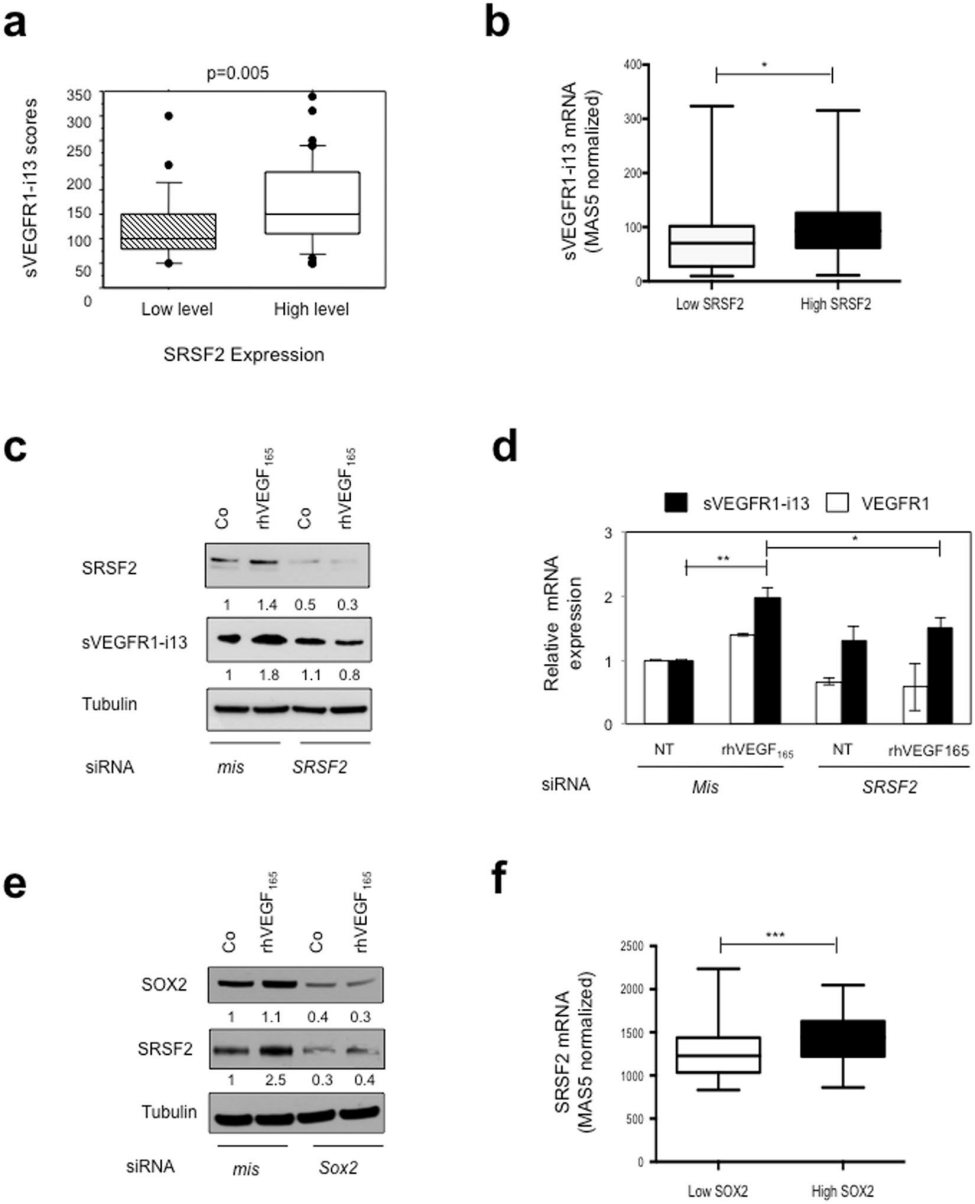
SRSF2 cooperates with SOX2 to regulate sVEGFR1-i13 expression in SQLC cells. (**a**) Mean levels ± SD of sVEGFR1-i13 immunohistochemical scores according to the SRSF2 status in squamous cell lung carcinoma, where SQLC are sub-divided in two classes representing tumors with high or low levels of SRSF2 compared to normal lung tissues^4,24^. (**b**) Mean levels ± SD of MAS5-normalized *sVEGFR1-i13* mRNA in SQLC patients taken from the GSE4573 database expressing either low (<50^th^ percentile) or high (>50^th^ percentile) levels of *SRSF2* mRNA. (**c**,**d**) MGH7 cells were transfected with *mismatch* (mis) or *Srsf2* (SRSF2) siRNA during 48 hours and treated or not (Co) for 24 additional hours with 1 ng/ml rhVEGF_165_. (**c**) Western blot analyses of SRSF2 and sVEGFR1-i13 proteins. Tubulin was used as a loading control. Numbers represent the quantification of SOX2, sVEGFR1-i13 or VEGF_165_ signal intensities using Image J software. The value 1 was arbitrarily assigned to the untreated condition signal. (**d**) RT-qPCR analyses of *sVEGFR1-i13* and *VEGFR1*. *GAPDH* was used as an internal control. (**e**) Western blot analyses of SOX2 and SRSF2 proteins in MGH7 cells transfected with *mismatch* (mis) or *Sox2* (Sox2) siRNA during 48 hours and treated or not (Co) for 24 additional hours with 1 ng/ml rhVEGF_165_. Tubulin was used as a loading control. Quantification as in (**c**). (**f**) Mean levels ± SD of MAS5-normalized *Srsf2* mRNA in SQLC patients taken from the GSE4573 database expressing either low (<50^th^ percentile) or high (>50^th^ percentile) levels of *Sox2* mRNA. Statistical analyses were performed using a non parametric Mann-Whitney test (*p < 0.05; **p < 0.01; ***p < 0.001). All western blot experiments were performed at least three times. Illustrations of a representative result are presented for each condition.

Then, we investigated the mechanism by which SOX2 controls *VEGFR1* pre-mRNA splicing. Serine Arginine Rich (SR) proteins belong to a family including twelve members that are critical splicing factors involved in constitutive and alternative pre-mRNA splicing^23^. We previously demonstrated that the SR proteins, SRSF1, SRSF2 and SRSF6, are up-regulated in NSCLC patients compared to normal lung tissues^24^. More recently, we observed an heterogeneous immunostaining of sVEGFR1-i13 in the same series of tumors^4^. Therefore, we looked for a putative relationship between sVEGFR1-i13 status and SR proteins levels. As shown in Fig. 3a, NSCLC patients with high sVEGFR1-i13 immunostaining scores were also those with high level of SRSF2 protein (p = 0.005). In contrast, no relationship was found between sVEGFR1-i13 scores and SRSF1 or SRSF6 status (patients with high versus low level; data not shown). To confirm these results, we took advantage of an Affymetrix dataset published in a cohort of 130 SQLC patients which contained two probe sets that distinguish between *sVEGFR1-i13* and full-length *VEGFR1* mRNAs (Gene Omnibus data set GSE4573^25^). We found that SQLC patients with high *SRSF2* mRNA level (>50^th^ percentile) also have high *sVEGFR1-i13* mRNA levels (Fig. 3b, p = 0.01). These results were confirmed by Spearman correlation analysis (Supplementary Fig. 2a, r = 0.5089, p < 0.0001). In contrast, no correlation was observed between *SRSF2* and *VEGFR1* mRNA levels (data not shown). To study whether SRSF2 was involved in the control of sVEGFR1-i13 by VEGF_165_, we transfected MGH7 cells with *mismatch* or *Srsf2* siRNA, and studied sVEGFR1-i13 expression by western blotting following rhVEGF_165_ treatment. The knock-down of SRSF2 significantly prevented the increase of sVEGFR1-i13 protein (Fig. 3c) or mRNA (Fig. 3d) levels induced by rhVEGF_165_. These data highly suggested that a VEGF_165_/SRSF2 network controls sVEGFR1-i13 expression in SQLC.

It was shown that the OSN (**O**CT4, **S**OX2, **N**ANOG) transcription complex controls the expression of SRSF2^26^. Therefore, we postulated that SRSF2 may be located downstream of the VEGF_165_/SOX2 network to regulate the expression of sVEGFR1-i13. In agreement with this hypothesis, the neutralization of SOX2 using siRNA prevented the SRSF2 accumulation in cells treated with rhVEGF_165_ (Fig. 3e). In addition, SQLC patients with high levels of *SOX2* mRNA (>50^th^ percentile) were also those with high *SRSF2* mRNA levels in the GSE4573 dataset (Fig. 3f, p = 0.0006). These results were confirmed by Spearman correlation analysis in the GSE4573 cohort (Supplementary Fig. 2b, r = 0.5342, p < 0.0001) as well as in another SQLC cohort (GSE68793; Supplementary Fig. 2c, r = 0.30, p = 0.0004). Of note, no significant correlation was observed between *SRSF2* and *SOX2* mRNA levels in two distinct lung ADC cohorts (Supplementary Fig. 2d,e). Taken together, these data demonstrated that a VEGF_165_/SOX2/SRSF2 signaling network controls sVEGFR1-i13 expression in SQLC patients.

### Anti-angiogenic therapies activate the VEGF_165_/SOX2/SRSF2 network to control sVEGFR1-i13 expression

**Figure 4:**
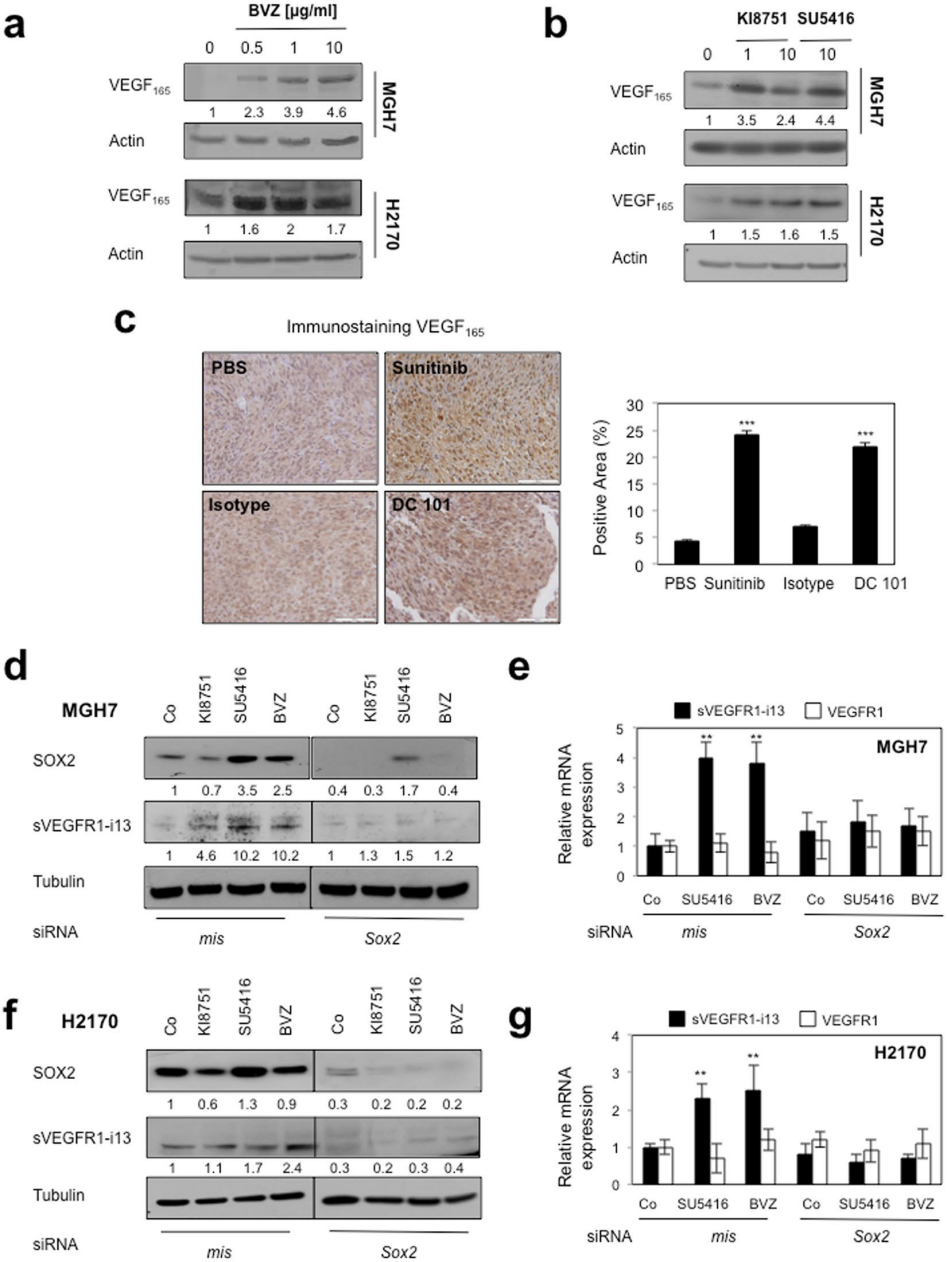
Anti-angiogenic therapies up-regulate sVEGFR1-i13 expression levels in SQLC cells through a SOX2-dependent mechanism. (**a**,**b**) MGH7 and H2170 cells were treated with the indicated concentrations of bevacizumab (µg/ml) for 72 hours (**a**) or KI8751 or SU5416 for 24 hours (**b**). Western blot experiments for the detection of VEGF_165_. Tubulin was used as a loading control. (**c**) Murine SQLC tumorgrafts having received sunitinib or the murine anti-VEGFR2 antibody DC101 or not (PBS, Isotype) were recovered from previous experiments^4,40^. Immunostaining of VEGF_165_ was performed. Right panels: automatic quantification of tumor cell immunostaining for each condition. Mean ± SD of 6 mice per condition. (**d**,**f**) Western blot experiments for the detection of the indicated proteins in MGH7 (**d**) or H2170 (**f**) cells transfected during 72 hours with either *mismatch* or *SOX2* siRNA and treated or not (Co) with 10 µg/ml bevacizumab, or transfected during 48 hours with either *mismatch* or *SOX2* siRNA and treated for 24 additional hours with 10 µM KI8751 or 10 µM SU5416. Tubulin was used as a loading control. Black delineations allow to separate differential parts of the same gel. Quantification and statistical analyses as described above. All western blot experiments were performed at least three times. Illustrations of a representative result are presented for each condition. (**e**,**g**) RT-qPCR analyses of *sVEGFR1-i13* or *VEGFR1* mRNA level were performed in cells treated in the same conditions as in d or f, respectively. *GAPDH* was used as an internal control. The value 1 was arbitrarily assigned to the untreated condition signal.

We recently demonstrated that anti-angiogenic therapies induce sVEGFR1-i13 expression (mRNA and protein) in squamous lung carcinoma cell lines and murine tumorgrafts^4^. Therefore, we asked whether the VEGF_165_/SOX2/SRSF2 network was involved. We first showed that bevacizumab, a monoclonal antibody targeting VEGF-A, or KI8751 or SU5416, two VEGFR tyrosine kinase inhibitors, induced the accumulation of intra-cellular VEGF_165_ protein in MGH7 and H2170 cells (Fig. 4a,b). The increase of intracellular VEGF_165_ was also observed in squamous lung carcinoma murine tumorgrafts treated with sunitinib, a VEGFR TKI, or DC101, an antibody against murine VEGFR2 (Fig. 4c). In these tumorgrafts, we previously showed an increase of sVEGFR1-i13 upon treatment with anti-angiogenic therapies^4^. Importantly, neutralization of SOX2 prevented the increase of intra-cellular sVEGFR1-i13 protein and mRNA levels in response to anti-angiogenic therapies in MGH7 (Fig. 4d,e) and H2170 cells (Fig. 4f,g). Conversely, the knock-down of SOX2 did not modify *VEGFR1* mRNA levels, whatever the treatments (Fig. 4e,g). *NOTCH1/NOTCH2* mRNA levels also did not vary upon anti-angiogenic treatments (Supplementary Fig. 1c), indicating that NOTCH signaling is not involved in the regulation of sVEGFR1-i13 expression in response to anti-angiogenic therapies. Of note, we were not able to test the effects of VEGF_165_ knock-down in these conditions as we did not find siRNAs selectively targeting VEGF_165_ only (data not shown).

**Figure 5:**
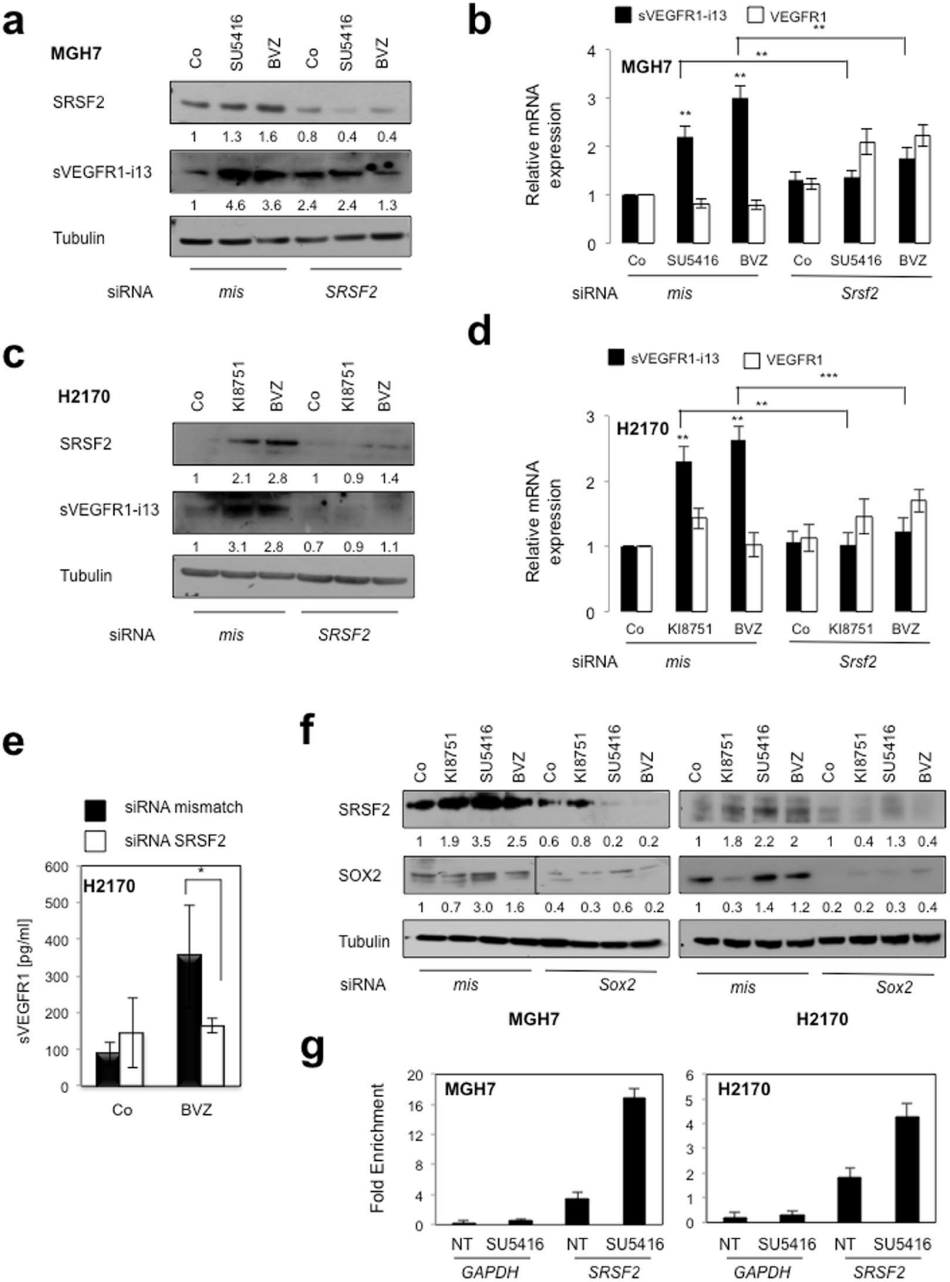
SOX2 and SRSF2 control sVEGFR1-i13 expression levels in response to anti-angiogenic therapies. MGH7 (**a**,**b**,**f**) or H2170 (**c**–**f**) cells were transfected during 72 hours with either *mismatch* or *Srsf2* (**a**–**e**) or *Sox2* (**f**) siRNA and treated or not (Co) with 10 µg/ml bevacizumab, or transfected during 48 hours with either *mismatch* or *Srsf2* (**a**–**e**) or *Sox2* (**f**) siRNA and treated for 24 additional hours with 10 µM KI8751 or 10 µM SU5416. (**a**,**c**,**f**) Western blot experiments for the detection of the indicated proteins. Tubulin was used as a loading control. Quantification (numbers) as previously described. Black delineation allows to separate differential parts of the same gel. All western blot experiments were performed at least three times. Illustrations of representative results are presented for each condition. (**b**,**d**) RT-qPCR analyses of *sVEGFR1-i13* or *VEGFR1* mRNA level were performed in cells treated in the same conditions as in a or c, respectively. *GAPDH* was used as an internal control. The value 1 was arbitrarily assigned to the untreated condition signal. (**e**) ELISA assays for quantification of sVEGFR1 protein level in the supernatants. Statistical analyses were performed using a non parametric Mann-Whitney test (*p < 0.05). (**g**) Chromatin immunoprecipitation experiments were performed using an anti-SOX2 (SOX2) or an irrelevant IgG (IgG) antibody. The genomic DNA regions encompassing two potential SOX2 binding sites of the *SRSF2* promoter were amplified by qPCR. The *GAPDH* promoter was used as a negative control. Results were normalized to input and expressed as fold enrichment compared with irrelevant antibody.

**Figure 6:**
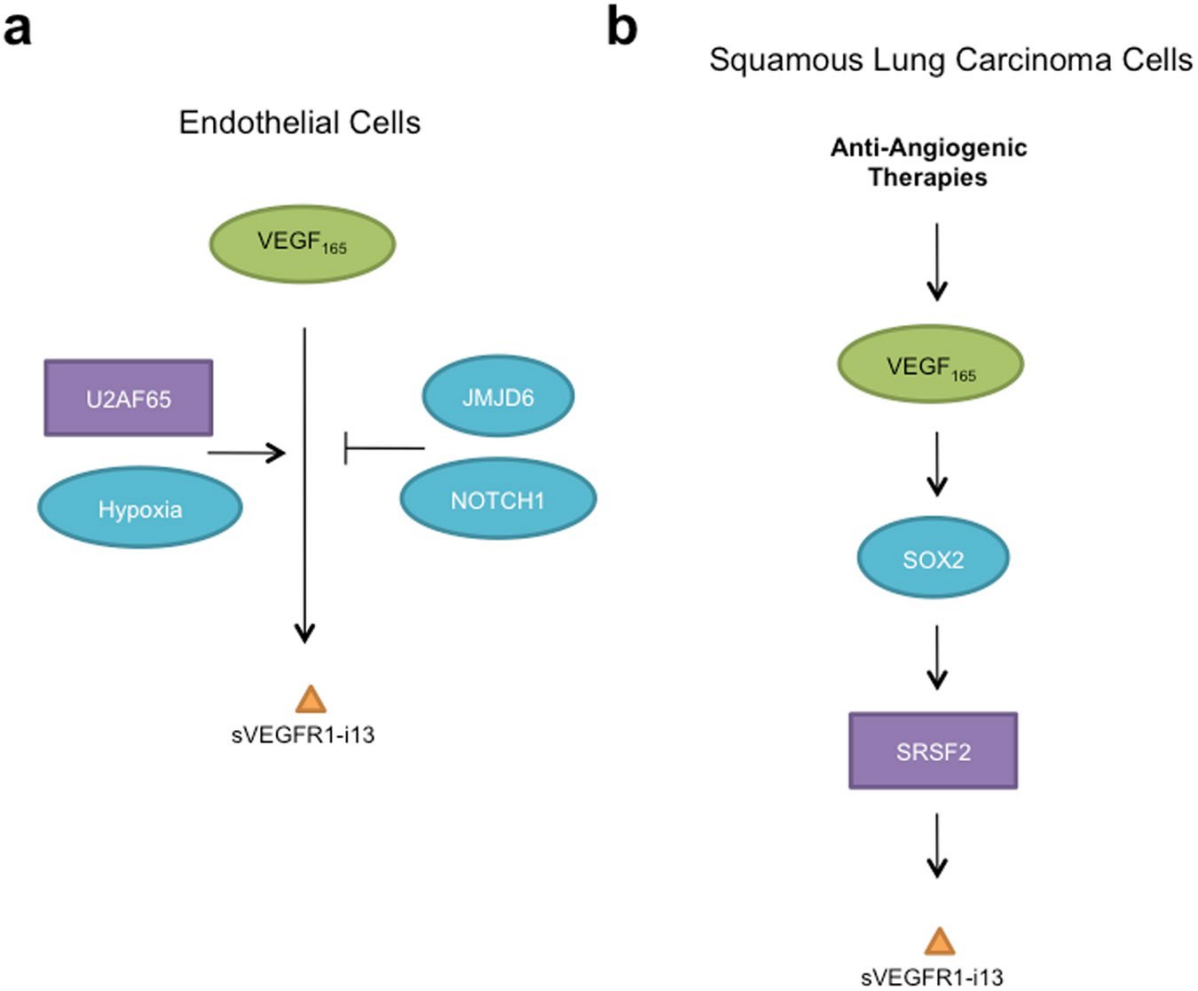
Molecular pathways that regulate sVEGFR1-i13 expression in endothelial and SQLC cells. (**a**) In endothelial cells, both positive (hypoxia, U2AF65 splicing factor) and negative (JMJD6, NOTCH1) regulators of sVEGFR1-i13 expression have been described in response to VEGF-A (VEGF_165_) stimulation. (**b**) In squamous lung carcinoma cells, anti-angiogenic therapies increase the intra-cellular level of VEGF_165_. VEGF_165_ induces the expression of the transcription factor SOX2 which controls SRSF2 protein level by binding to the *SRSF2* promoter. As a final step, SRSF2 controls the alternative splicing of *VEGFR1* towards *sVEGFR1-i13* expression.

Moreover, in both cell lines, the accumulation of sVEGFR1-i13 following anti-angiogenic treatments was associated with an increase of SRSF2 protein level and was significantly prevented in cells deprived of SRSF2, both at the protein (Fig. 5a,c,e) and mRNA (Fig. 5b,d) levels. The knock-down of SRSF2 did not modulate *VEGFR1* mRNA levels, whatever the conditions. Furthermore and consistent with a role of SOX2 in regulating SRSF2 expression, SOX2 knock-down prevented SRSF2 accumulation in response to anti-angiogenics in both cell lines (Fig. 5f). More importantly, by performing chromatin immunoprecipitation experiments, we finally showed that SOX2 directly binds to the *SRSF2* promoter and that SU5416 treatment increases this binding (Fig. 5g). As a whole, these data demonstrated that a VEGF_165_/SOX2/SRSF2 network controls *VEGFR1* pre-mRNA splicing towards sVEGFR1-i13 expression in lung cancer cells treated with anti-angiogenic therapies (Fig. 6).

## Discussion

To our knowledge, nothing is known regarding the molecular mechanisms that regulate the expression of VEGFR1 splice variants in cancerous cells. Hence, high levels of sVEGFR1s have been previously reported in plasma, serum or tissues of many types of cancer such as colorectal cancer, breast cancer, glioblastoma and lung cancer^27–30^. Such increase has been mainly correlated with poor prognosis, but the molecular mechanisms behind this regulation have not been clearly elucidated. In addition, several clinical trials have reported variation in circulating levels of sVEGFR1 following anti-angiogenic therapies, and a high level of sVEGFR1s was correlated with a poor therapeutic response^5–7,31–35^. Again, none of these studies has investigated intra-tumoral levels of sVEGFR1s, nor pointed out to a a specific mechanism for sVEGFR1 generation. Recently, we demonstrated that the sVEGFR1-i13 splice variant is up-regulated in lung cancer patients, and contributes to the progression and the escape of squamous lung carcinoma from anti-angiogenic therapies^4^. In this study, we demonstrate that a signaling network involving the VEGF_165_, SOX2 and SRSF2 proteins controls the expression of sVEGFR1-i13 in these tumors. More importantly, we demonstrate that this signaling network also controls the expression of sVEGFR1-i13 in response to anti-angiogenic therapies. These results identify for the first time upstream regulators of *VEGFR1* pre-mRNA splicing in cancer cells.

In endothelial cells, the expression of sVEGFR1-i13 is up-regulated by VEGF-A^15,16^, hypoxia^36^ or by decreased expression of the oxygen-sensing hydroxylase JMJD6 which controls the hydroxylation of the splicing factor U2AF65^17,37^. Beside VEGF-A, we also studied the impact of hypoxia on sVEGFR1-i13 expression and the status of JMJD6 in our cellular models, but we did not find any significant effect in cells treated or not with anti-angiogenics (data not shown). We focused on SOX2 because its amplification occurs in about 30% of SQLC patients^21^, and because VEGF-A was shown to control SOX2 expression in cancer cells^20^. In this study, we identified SOX2 as an upstream regulator of sVEGFR1-i13 in SQLC cells. In addition, we showed that SOX2 regulates the expression of the SR protein SRSF2, an ubiquitous splicing factor that plays a critical role in both constitutive and alternative pre-mRNA splicing^23^. We previously demonstrated that SRSF2 protein is overexpressed in SQLC patients as compared to normal lung tissues^24^. In this study, we observed an association between SRSF2 and sVEGFR1-i13 protein levels in the same series of patients (Fig. 3a, p = 0.005). Interestingly, SRSF2 was recently shown to be part of a WNT5a-dependent signaling network controlling sVEGFR1 expression in aged mice exposed to ischemic stress^38^. In addition, we found a correlation between *SRSF2* and *SOX2* mRNA levels in two Gene Omnibus cohorts, namely GSE4573 (Supplementary Fig. 2b, p < 0.0001) and GSE68793 (Supplementary Fig. 2c, p = 0.0004). This was consistent with SOX2 and SRSF2 being closely connected in SQLC tumors. In agreement, we showed that SRSF2 is a direct target gene of SOX2 in SQLC cell lines. SOX2, together with OCT4 and NANOG, is part of the transcription OSN complex, and SRSF2 is an OCT4 target gene required for pluripotency in human pluripotent stem cell (hPSC)^39^. Therefore, these and our results highly suggest that SRSF2 could be an important mediator of the OSN complex function in non transformed and transformed cells. In this setting, it remains to characterize the upstream signals that control the activation of the VEGF_165_/SOX2/SRSF2 pathway in lung tumors. We recently observed a correlation between sVEGFR1-i13 and β1 integrin expression in SQLC cell lines and primary tumors^4^. Therefore, one possibility is that β1 integrin senses the extracellular matrix to activate the VEGF_165_/SOX2/SRSF2 pathway. We are currently testing this hypothesis.

We found that sVEGFR1-i13 accumulates in SQLC cells in response to anti-angiogenic therapies by a mechanism that requires SOX2 and SRSF2 proteins. In pre-clinical SQLC murine models, resistance of tumor cells to anti-angiogenic therapies has been associated with the accumulation of Cancer Stem Cells markers (CSC)^40^. In addition, it was previously shown that VEGF-A and SOX2 proteins cooperate to promote CSC self-renewal in lung cancer cells^20^ and that SRSF2 is the most enriched splicing factor in human pluripotent stem cells^26^. It has been suggested that high levels of sVEGFR1 contribute to tumor escape from anti-angiogenic therapies by decreasing baseline microvascular density^32,34^. Based on our results, it is thus tempting to speculate that high levels of sVEGFR1-i13 may also be part of a VEGF/SOX2/SRSF2 axis involved in CSC self-renewal post-treatment.

To conclude, we highlight VEGF-A, SOX2 and SRSF2 proteins as regulators of VEGFR1 pre-mRNA alternative splicing in SQLC, and demonstrate that anti-angiogenic therapies affect this network. Because treatment of SQLC patients remains very challenging, our data offer a new signaling pathway to be explored in these patients for potential therapeutic avenues.

## Materials and Methods

### Cells, cell culture and reagents

MGH7 and H2170 squamous lung carcinoma cell lines were cultured in 5% CO_2_ at 37 °C in RPMI-1640 medium supplemented with 2 mM L-glutamine and 10% (v/v) Fetal Calf Serum (FCS) as previously described^41^. Bevacizumab (Avastin®) was kindly provided by Roche/Genetech, Indianapolis, USA. VEGFR2 kinase inhibitor KI8751 (cat#676484) was from Calbiochem. SU5416 (cat#S8442) and the inhibitor of Notch signaling FLI-06 (cat#SM0975) were purchased from Sigma-Aldrich. The human recombinant VEGF-A ligands rhVEGF_165_ (cat#293-VE) and rhVEGF_121_ (cat#4644-VS) were supplied by R&D Systems, whereas rhVEGF_189_ (cat#CRV114A) was from Cell Sciences (Canton, USA). The plasmids used in this study were: pcDNA3.1, pcDNA3.1-VEGF_165_ (kindly provided by Pr David Bates, University of Notthingham, UK). Transfections of plasmid DNA were performed using X-tremeGENE 9 (Roche), according to the manufacturer’s instructions. Cells were analyzed 48 h after transfection. All methods were performed in accordance with the relevant guidelines and regulations.

### Patients, tissue and subcutaneous tumorgrafts samples

Seventy-seven human NSCLC and 17 matched normal lung parenchymas were included in this study. Tumors consisted of 41 lung adenocarcinoma (ADC) and 36 SQLC. Tumor tissues and normal lung parenchyma, taken away from the bulk of the tumor, were collected from resection of lung tumors, and stored for scientific research in a biological resource repository (Centre de Ressources Biologiques, CHU Albert Michallon, Grenoble Hospital) following national ethical guidelines. All patients enrolled in this trial provided written informed consent. Tissue banking and research conduct was approved by the Ministry of Research (approval AC-2010-1129) and by the regional IRB (CPP 5 Sud Est). For histological classification, tumor samples were fixed in formalin, and diagnosis was made on paraffin-embedded material using the WHO VII^th^ classification of lung criteria^42^. For each case, one section from the most representative block was chosen. These sections always contained more than 70% tumor cells. Immunohistochemical stainings of VEGF_165_ and sVEGFR1-i13 were performed as previously described^4^. Sections from UN-SCC680 subcutaneous tumorgrafts were recovered from previous experiments^40^ and stained for VEGF_165_. For automatic quantification of VEGF_165_ staining in mouse models, sections were scanned using a ZEISS AxioImager M2 automated slide scanner with 5X magnification and the images were analyzed using Image J software. Threshold values were adjusted until masked brownpixels correlated with positive immunostaining or with total area of the digitized tissue. The percentage of positive areas was then calculated for each staining. The Affymetrix datasets GSE4573 published in a cohort of 130 SQLC patients and containing two probe sets that distinguish between *sVEGFR1-i13* and full-length *VEGFR1* mRNAs^25^ and GSE68793 were recovered from Gene Omnibus (GEO).

### RNA interference

The two siRNA specifically targeting sVEGFR1-i13 were: sVEGFR1-i13(1) sense, 5′-UAA-CAG-UUG-UCU-CAU-AUC-3′; anti-sense, 5′-UGA-UAU-GAG-ACA-ACU-GUU-A-3′ and sVEGFR1-i13(2) sense, 5′-UCU-CGG-AUC-UCC-AAA-UUU-3′; anti-sense, 5′-UAA-AUU-UGG-AGA-UCC-GAG-A-3′. The sequences for SOX2 siRNA were designed as sense, 5′-AAAGGCUGGAAGUCAGCACUAAUUU-3′; and anti-sense, 5′-AAAAAUUAGUGCUGACUUCCAGCUU-3′. The sequences for SRSF2 were designed as SRSF2 (1) sens5′-GAG-GAC-GCU-AUG-GAU-GCC-AUG-GAC-G55-3′; anti-sens, 5′-CGU-CCA-UGG-CAU-CCA-UAG-CGU-CCU-C55-3′ and SRSF2 (2) sens, 5′-UCG-AAG-UCU-CGG-UCC-CGC-ACU-CG5-5-3′; anti-sens, 5′-CGA-GUG-CGG-GAC-CGA-GAC-UUC-GA5-5-3′. Transfection of siRNA oligonucleotide duplexes was performed using JetPrime reagent (Ozyme, Saint Quentin en Yvelines, France) for MGH7 cells and RNAi max (Invitrogen) for H2170 cells according to the manufacturer’s protocol. The scrambled siRNA oligonucleotides used as a control for all RNA interference experiments were as follows: forward 5′-UCGGCUCUUACGCAUUCAATT-3′ and reverse 5′-CAAGAAAGGCCAGUCCAAGTT-3′. Cells were analysed 72 hours post-transfection.

### ELISA assays

ELISA assays were performed in duplicate in 96-wells plates using a Quantikine sVEGFR1 kit (R&D Systems). Manipulations were carried out according to manufacturer’s instructions. Briefly, 1.5 × 10^6^ cells/well were seeded in 6-wells plates and treated or not for different times. The concentration of sVEGFR1 in the supernatants was calculated from the absorbance value compared to the standard curve and expressed in pg/ml.

### RNA extraction, reverse transcription and real-time qPCR analysis

Total RNA was extracted using the High Pure RNA Isolation Kit (Roche Diagnostics) according to the manufacturer’s protocol. RNA concentration and integrity was determined using a NanoDrop ND-1000 spectrophotometer (Labtech). Then, one microgram of total RNA was subjected to Reverse Transcription using iScript RT supermix (Bio-Rad, Marnes-la-Coquette, France). Quantitative RT-PCR (qRT-PCR) was performed using iTaq® qPCR Universal SYBR Green Supermix (Bio-Rad). The primer sequences used were as follows: *sVEGFR1-i13*: 5′-AGGGGAAGAAATCCTCCAGA-3′(forward) and 5′-CAACAAACACAGAGAAGG-3′(reverse); *VEGFR1(1)*: 5′-AGGGGAAGAAATCCTCCAGA-3′ (forward) and 5′-CGTGCTGCTTCCTGGTCC-3′(reverse); *VEGFR1(2):* 5′-ACCGAATGCCACCTCCATG-3′(forward) and 5′-AGGCCTTGGGTTTGCTGTC-3′(reverse); *NOTCH-1:* 5′-GCCGCCTTTGTGCTTCTGTTC-3′(forward) AND 5′-CCGGTGGTCTGTCTGGTCGTC-3′(reverse); *NOTCH-2:* 5′-GCCTTGCTGAAGACAGGAAG-3′(forward) and 5′-CAACTGCCTCTGCACAATGA-3′(reverse); *SOX2*: 5′-TGATGGAGACGGAGCTGAA-3′(forward) and 5′-GGGCTGTTTTTCTGGTTGC-3′(reverse); *GAPDH*: 5′-CGAGATCCCTCCAAAATCAA-3′(forward) and 5′-ATCCACAGTCTTCTGGGTGG-3′(reverse). Relative gene expression was calculated, for each sample, as the ratio of specific target gene to GAPDH gene (reference gene), thus normalizing the expression of target gene for sample to sample differences in RNA input.

### Antibodies and immunoblotting

Immunoblotting experiments were performed as previously described^43^. The antibodies used were: anti-actin from Sigma, anti-tubulin (clone B512, sc-23948) from Santa Cruz, anti-SOX2 (AB5603) and anti-phospho-VEGFR1-Tyr1213 (cat#07-758) from Millipore and anti-SRSF2 (clone 4F-11) from Euromedex. The specific anti-sVEGFR1-i13 was generated against a peptide mapping in the unique C-terminus^13^. We previously checked that this antibody recognizes sVEGFR1-i13 protein in our cells by using siRNA targeting retained intron 13 in sVEGFR1-i13^4^. The anti-VEGF_165_ antibody raised against the six terminal amino acids (CDKPRR sequence) and a sixteen amino acids sequence targeting terminal part of VEGF_165_ encompassing exons 7 and 8a was produced by Covalab (Villeurbanne, France). We previously checked that this antibody recognizes a recombinant VEGF_165_ but not VEGF_165_b protein^19^.

### Chromatin immunoprecipitation assay

Chromatin immunoprecipitation experiments were performed in MGH7 and H2170 cells treated or not (NT) with 10 µM SU5416 for 24 hours. ChIP experiments were performed using the ChIP-IT^R^ Express Magnetic Chromatin Immunoprecipitation kit from Active Motif (La Hulpe, Belgium) according to manufacturer’s instructions. Briefly, cells were formaldehyde cross-linked and chromatin was isolated and sonicated using a Bioruptor apparatus. An equal amount of chromatin (30 µg) was precleared, immunoprecipitated with either a polyclonal antibody specific for SOX2 (D6D9 XP^R^, ChIP formulated, Cell Signaling) or unrelated rabbit IgG, overnight at +4 °C, washed and reverse cross-linked. One-twentieth of the immunoprecipitated chromatin was analyzed for the presence of *SRSF2* promoter DNA by Q-PCR using primers that flanked two potential SOX2 consensus binding sites (TTGT) at (−169; −165) and (−216; −212) positions on the promoter. A sequence corresponding to the *GAPDH* promoter was used as a negative control in SOX2 ChIP. Q-PCR studies were performed using using iTaq® qPCR Universal SYBR Green Supermix (Bio-Rad). Input DNA sample corresponding to 1% of immunoprecipitated chromatin was analyzed in parallel in order to normalized the results of each ChIP DNA sample to the corresponding input DNA sample. The primers used were as follow: *SRSF2* forward 5′-AAGGTTTCATTTCCGGGTGG-3′; *SRSF2* reverse 5′-GGGACACTGGGAAAGGCCA-3′; *GAPDH* forward 5′-AGCTCAGGCCTCAAGACCTT-3′ and *GAPDH* reverse 5′-AAGAAGATGCGGCTGACTGT-3′.

### Statistical analyses

The statistical analyses were performed using Statview software (Abacus Concepts). Descriptive analyses comparing continuous and two-level categorical variables were carried out using the Mann-Whitney U test. P values < 0.05 were considered significant.

## Electronic supplementary material

Supplementary datasets

## Data Availability

All data generated or analysed during this study are included in this published article (and its Supplementary Information Files).
