# Supplementary material for: A VEGF-A/SOX2/SRSF2 network controls VEGFR1 pre-mRNA alternative splicing in lung carcinoma cells
Source: Sci Rep. 2019 Jan 23;9:336. doi: 10.1038/s41598-018-36728-y (PMC6344584; doi:10.1038/s41598-018-36728-y)
Supplement: Supplementary file 1 — Supplementary datasets [file 41598_2018_36728_MOESM1_ESM.pdf]

A VEGF-A/SOX2/SRSF2 network controls *VEGFR1* pre-mRNA alternative splicing in lung carcinoma cells

Abou Fayçal Cherine[1,2], Gazzeri Sylvie[1,2] and Eymin Beatrice[1,2*]

[1] INSERM U1209, CNRS UMR5309, Institute For Advanced Biosciences, Grenoble, 38042, France.

[2] Université Grenoble Alpes, Institut Albert Bonniot, Grenoble, 38041, France.

Supplementary Information

Supplementary Figure legend

Supplementary Figure 1. Inhibition of NOTCH signaling does not affect sVEGFR1-i13 level in SQLC cells. (a) MGH7 and H2170 cells were treated with 10μM FLI-O6 for 24 additional hours. Western blot analysis of sVEGFR1-i13 as indicated. Tubulin was used as a loading control. Numbers represent the quantification of signal intensities for each protein using Image J software. The value 1 was arbitrarily assigned to the untreated condition signal. (b) MGH7 or H2170 cells were transfected during 48 hours with either *mismatch* or *sVegfr1-i13* siRNA and treated for 24 additional hours with 10μM FLI-06. RT-qPCR analyses to quantify *sVEGFR1-i13* (black histograms) and *VEGFR1* (white histograms) mRNA levels. *GAPDH* was used as an internal control. (c) MGH7 (left panel) or H2170 (right panel) cells were treated with 10μg/ml bevacizumab for 72 hours or 10μM KI8751 or SU5416 for 24 hours. RT-qPCR analyses to quantify *Notch1* (black histograms) and *Notch2* (white histograms) mRNA levels. *GAPDH* was used as an internal control. Statistical analyses were performed using a non parametric Mann-Whitney test.

Supplementary Figure 2. *SRSF2* and *SOX2* mRNA levels are correlated in SQLC but not in lung ADC patients. (a) Spearman correlation analysis of *SRSF2* and *sVEGFR1-i13* mRNA levels in SQLC patients using the GSE4573 GEO dataset. (b) Spearman correlation analysis of *SOX2* and *SRSF2* mRNA levels in SQLC patients using the GSE4573 GEO dataset. (c) Spearman correlation analysis of *SRSF2* and *SOX2* mRNA levels in SQLC patients using the GSE68793 GEO dataset. (d, e) Spearman correlation analysis of *SRSF2* and *SOX2* mRNA levels in lung ADC patients using two distinct GEO datasets, TCGA (d) or GSE68465 (e). p and r values are indicated in each case. NS: not significant.

a

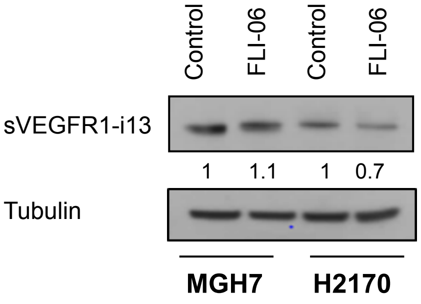
Control
FLI-06
Control
FLI-06
sVEGFR1-i13
1 1.1 1 0.7
Tubulin
MGH7
H2170

b

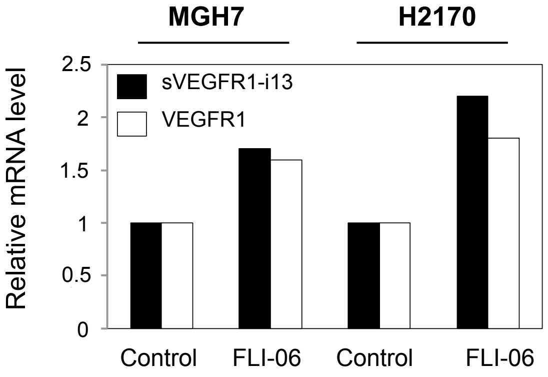
MGH7
H2170
Relative mRNA level
sVEGFR1-i13
VEGFR1
2.5
2
1.5
1
0.5
0
Control
FLI-06
Control
FLI-06

c

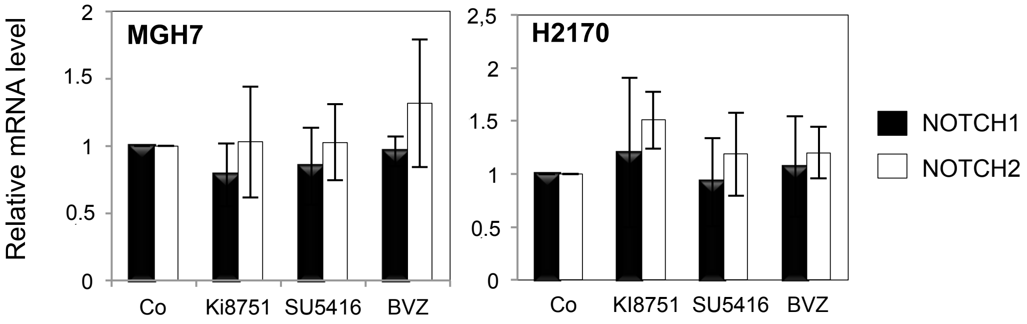
Relative mRNA level
MGH7
2
1.5
1
0.5
0
Co
Ki8751
SU5416
BVZ
H2170
2,5
2
1.5
1
0.5
0
Co
KI8751
SU5416
BVZ
NOTCH1
NOTCH2

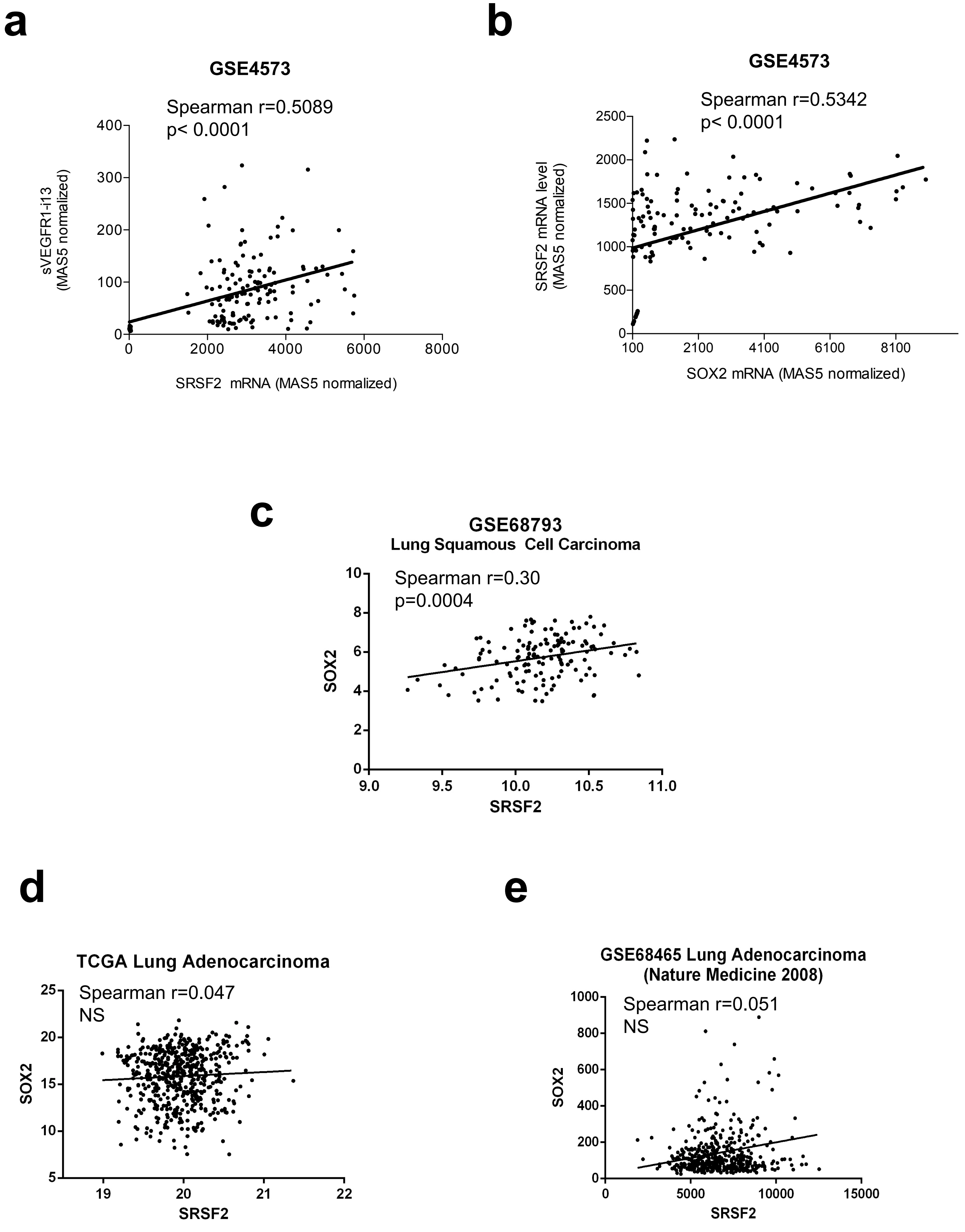
a
GSE4573
Spearman r=0.5089
p< 0.0001
sVEGFR1-i13
(MAS5 normalized)
SRSF2 mRNA (MAS5 normalized)
b
GSE4573
Spearman r=0.5342
p< 0.0001
SRSF2 mRNA level
(MAS5 normalized)
SOX2 mRNA (MAS5 normalized)
c
GSE68793
Lung Squamous Cell Carcinoma
Spearman r=0.30
p=0.0004
SOX2
SRSF2
d
TCGA Lung Adenocarcinoma
Spearman r=0.047
NS
SOX2
SRSF2
e
GSE68465 Lung Adenocarcinoma
(Nature Medicine 2008)
Spearman r=0.051
NS
SOX2
SRSF2

Original Blots

Figure 1e

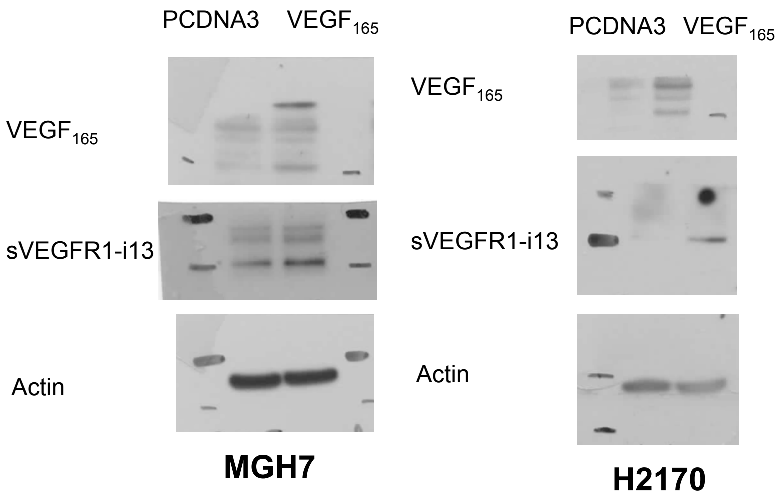

Figure 2a

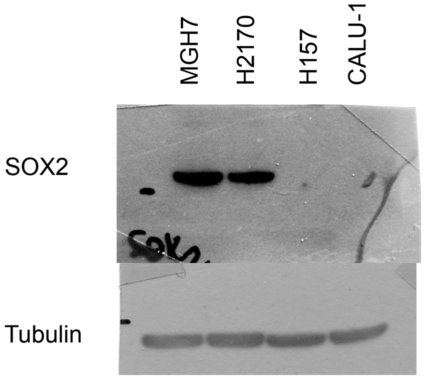

Figure 2b

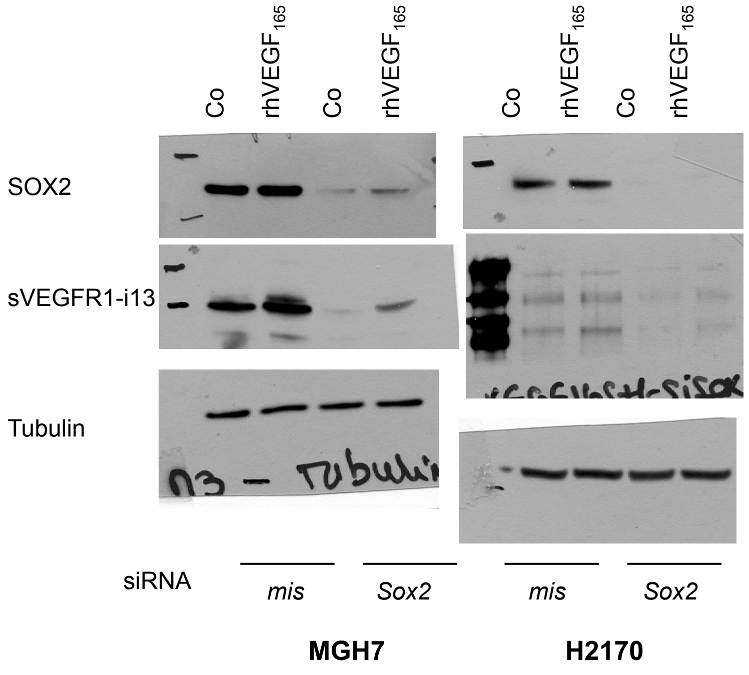

Figure 2d

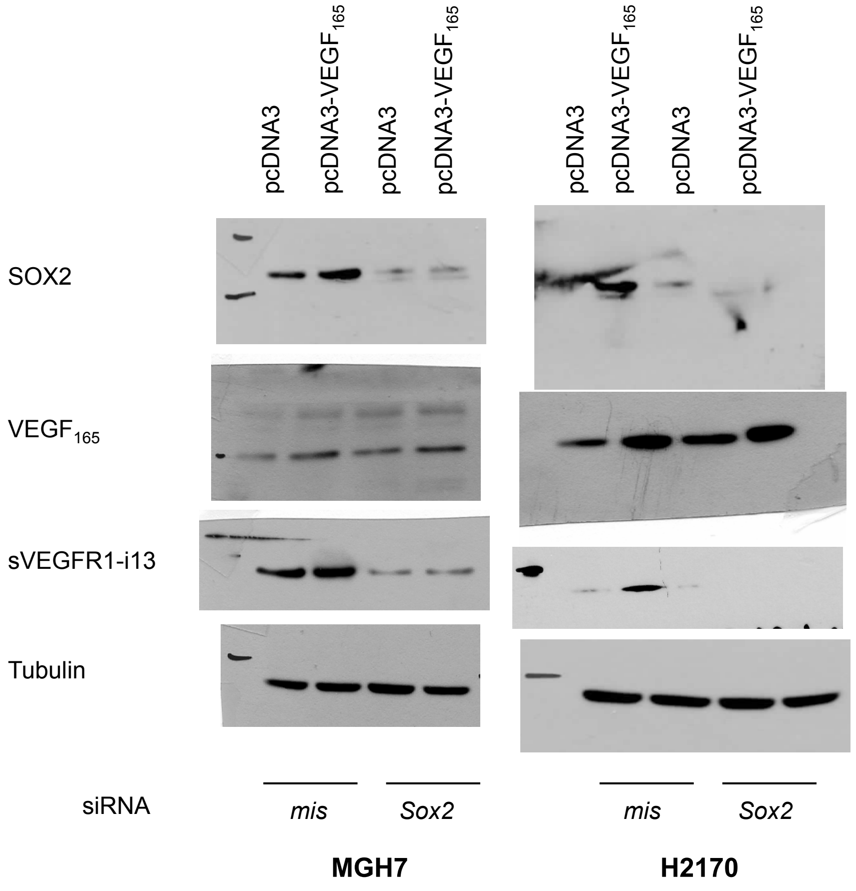
pcDNA3
pcDNA3-VEGF165
pcDNA3
pcDNA3-VEGF165
pcDNA3
pcDNA3-VEGF165
pcDNA3
pcDNA3-VEGF165
SOX2
VEGF165
sVEGFR1-i13
Tubulin
siRNA
mis
Sox2
mis
Sox2
MGH7
H2170

Figure 3c

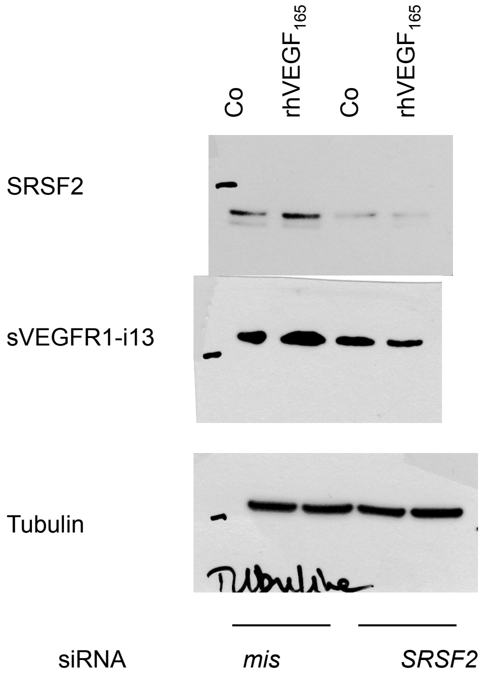

Figure 3e

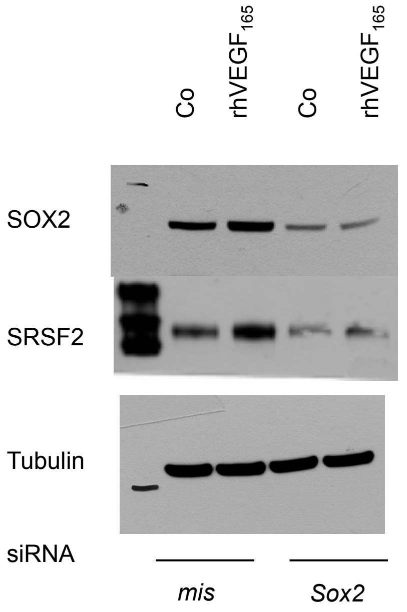

Figure 4a

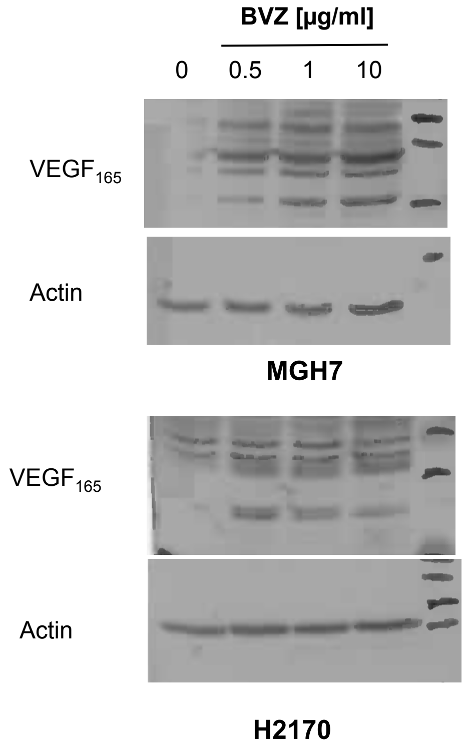

Figure 4b

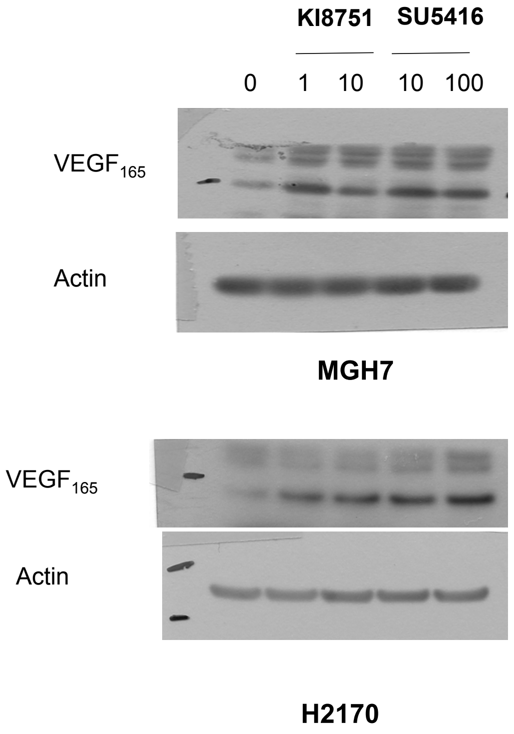

Figure 4d

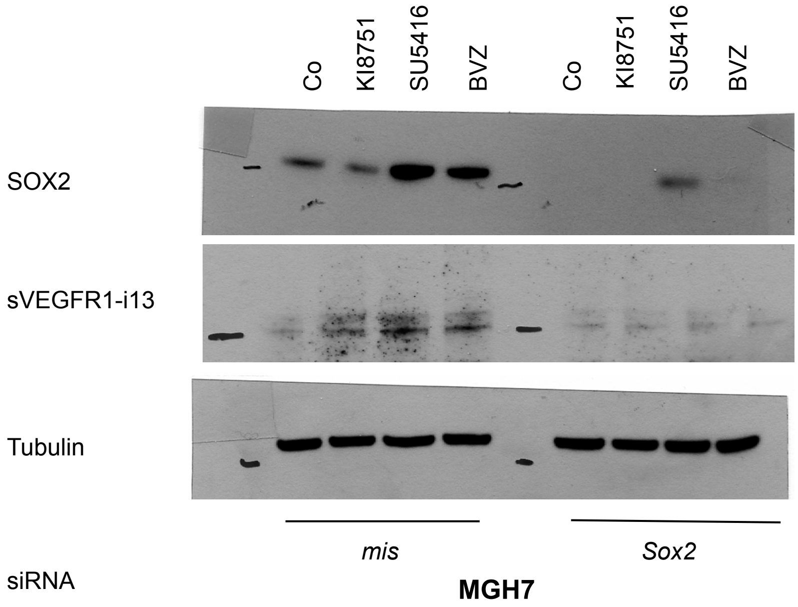
Co
KI8751
SU5416
BVZ
Co
KI8751
SU5416
BVZ
SOX2
sVEGFR1-i13
Tubulin
siRNA
mis
Sox2
MGH7

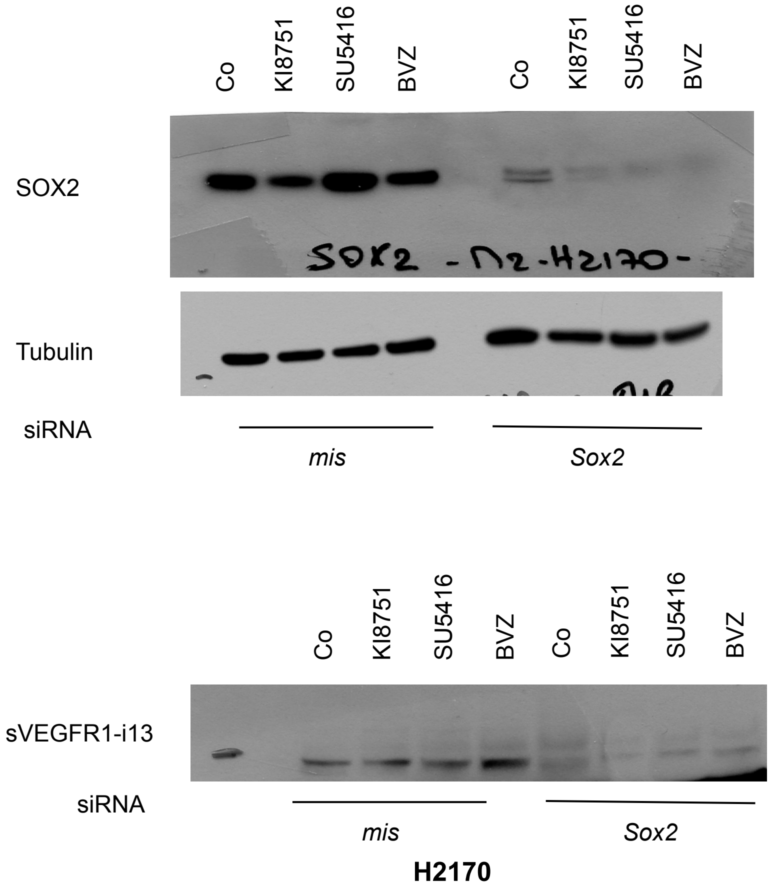
Co
KI8751
SU5416
BVZ
Co
KI8751
SU5416
BVZ
SOX2
Tubulin
siRNA
mis
Sox2
Co
KI8751
SU5416
BVZ
Co
KI8751
SU5416
BVZ
sVEGFR1-i13
siRNA
mis
Sox2
H2170

Figure 5a

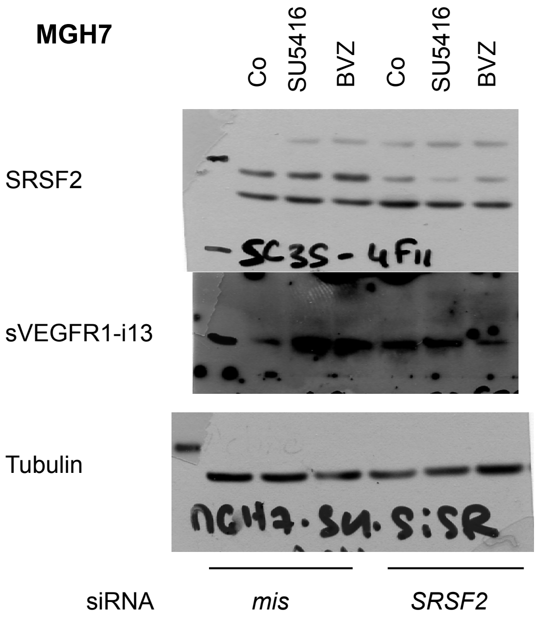

Figure 5c

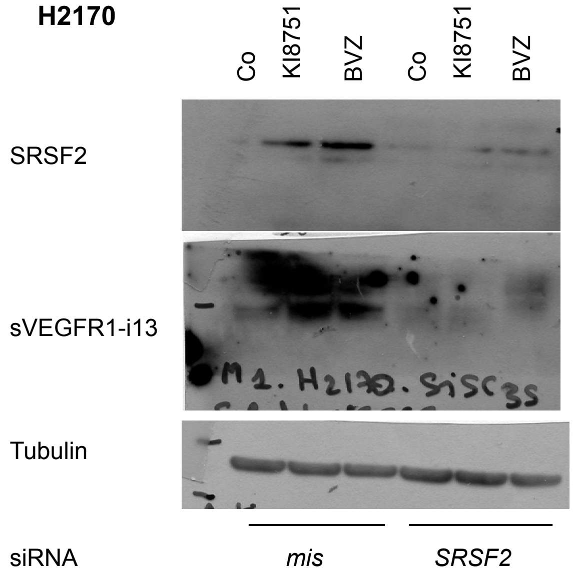

Figure 5f

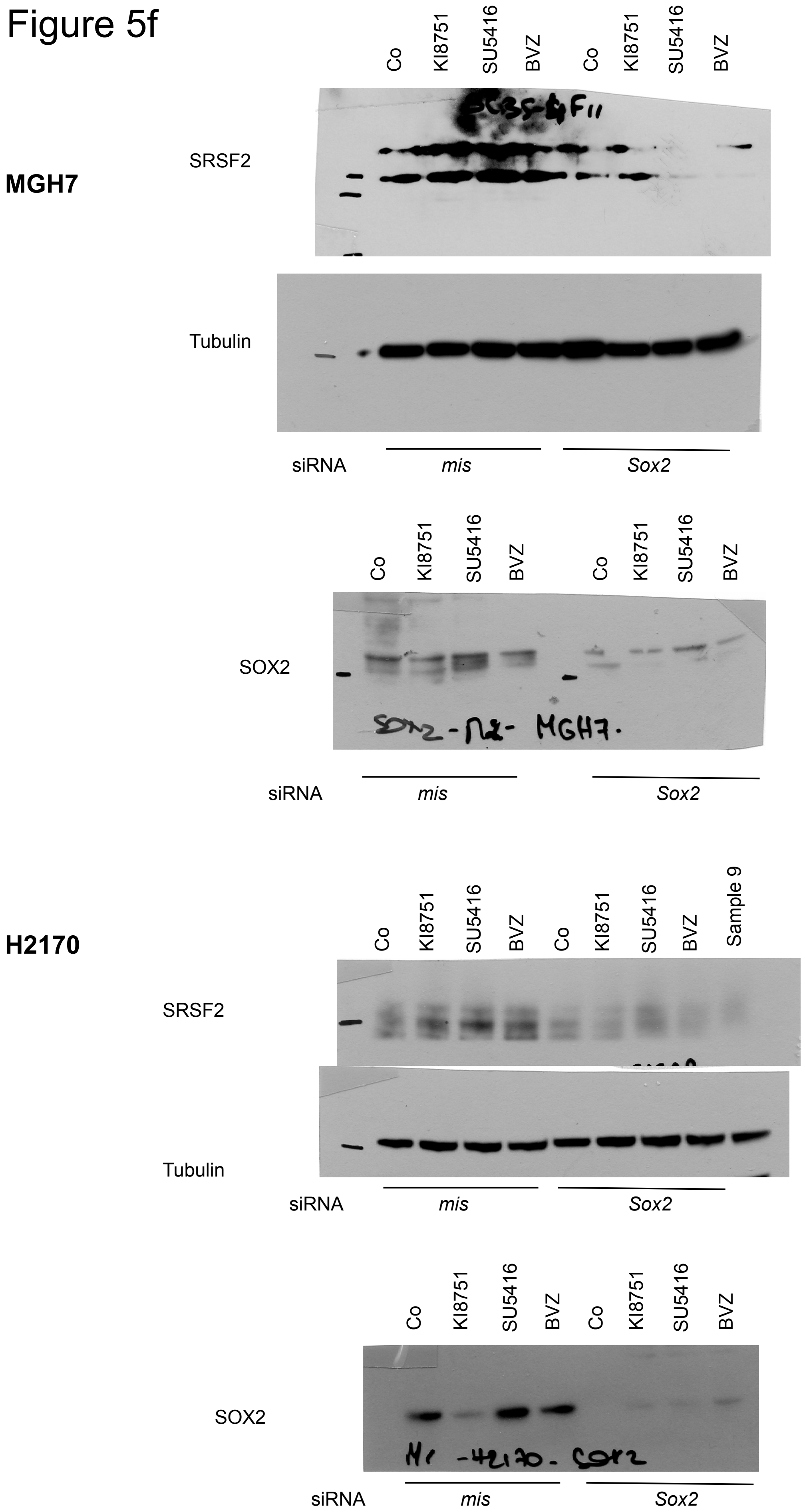
MGH7
Co
KI8751
SU5416
BVZ
Co
KI8751
SU5416
BVZ
SRSF2
Tubulin
siRNA
mis
Sox2
Co
KI8751
SU5416
BVZ
Co
KI8751
SU5416
BVZ
SOX2
siRNA
mis
Sox2
H2170
Co
KI8751
SU5416
BVZ
Co
KI8751
SU5416
BVZ
Sample 9
SRSF2
Tubulin
siRNA
mis
Sox2
Co
KI8751
SU5416
BVZ
Co
KI8751
SU5416
BVZ
SOX2
siRNA
mis
Sox2
